# Efficacy and Safety of Medicinal Plants or Related Natural Products for Fibromyalgia: A Systematic Review

**DOI:** 10.1155/2013/149468

**Published:** 2013-06-04

**Authors:** Simone de Souza Nascimento, Josimari Melo DeSantana, Fernando Kenji Nampo, Êurica Adélia Nogueira Ribeiro, Daniel Lira da Silva, João Xavier Araújo-Júnior, Jackson Roberto Guedes da Silva Almeida, Leonardo Rigoldi Bonjardim, Adriano Antunes de Souza Araújo, Lucindo José Quintans-Júnior

**Affiliations:** ^1^Department of Physiology, Federal University of Sergipe, Marechal Rondom Avenue, 49000-100 São Cristovão, SE, Brazil; ^2^Collegiate of Physical Therapy, Federal University of Sergipe, Cláudio Batista Street, 49060-100 Aracaju, SE, Brazil; ^3^Department of Nursing and Pharmacy, Federal University of Alagoas, Lourival de Mello Motta Avenue, 57072-970 Maceió, AL, Brazil; ^4^Department of Pharmaceutical Sciences, Federal University of São Francisco Valley, José de Sá Maniçoba Avenue, 56304205 Petrolina, PE, Brazil; ^5^Department of Biological Sciences, Bauru School of Dentistry, University of São Paulo, Octávio Pinheiro Brisola Street, 17012-901 Bauru, SP, Brazil

## Abstract

To assess the effects of medicinal plants (MPs) or related natural products (RNPs) on fibromyalgia (FM) patients, we evaluate the possible benefits and advantages of MP or RNP for the treatment of FM based on eight randomized placebo-controlled trials (RCTs) involving 475 patients. The methodological quality of all studies included was determined according to JADAD and “Risk of Bias” with the criteria in the Cochrane Handbook for Systematic Reviews of Interventions 5.1.0. Evidence suggests significant benefits of MP or RNP in sleep disruption, pain, depression, joint stiffness, anxiety, physical function, and quality of life. Our results demonstrated that MP or RNP had significant effects on improving the symptoms of FM compared to conventional drug or placebo; longer tests are required to determine the duration of the treatment and characterize the long-term safety of using MP, thus suggesting effective alternative therapies in the treatment of pain with minimized side effects.

## 1. Introduction

Fibromyalgia (FM) is an example of rheumatologic disease that leads to chronic musculoskeletal pain diffuse, usually associated to fatigue, sleep disruption, mood disorders, and depression, among other symptoms. Although its pathogenesis is not fully understood, most of the evidence points to either a disorder in the central pain modulation or an alteration in the processing of the central nervous system in response to a noxious stimulus [1–5].

Many therapeutic approaches are sought by patients to control symptoms of FM. Pharmacologic therapy is one of the resources used in the treatment of chronic pain. Among the most widely used drugs are opioids, nonsteroidal anti-inflammatory drugs (NSAIDs), muscle-relaxing medications, painkillers, and peripheral antidepressants [6]. However, 40 to 60% of patients do not respond well to drug therapy [7, 8]. For this reason, the search for novel therapies remains constant throughout the world [9].

A current approach is to develop a new biological compound from natural products that inhibits pain, such as MP or its secondary metabolites, with enhanced efficacy and minimal side effects [10, 11]. Butler (2008) [11] conducted an extensive research on the drugs approved by the Food and Drug Administration (FDA, USA) in the last decade and described that about 40% of new drugs originate from natural products. Although the application of medicinal plants in treatments around the world has suffered a strong advance, there are still a few studies that prove the safe and effective use of various plants [12]. Notable progress has been made in the recent years in the development of natural therapies, but there is an urgent need to discover effective and potent analgesic agents [13]. 

As the evidence-gathering tools, systematic reviews (SRs) of randomized controlled trials (RCTs) are considered to provide the best evidence about the effectiveness of interventions [14]. Therefore, the aim of this systematic review was to examine the clinical evidence on the use of medicinal plants for FM based on RCTs of herbal preparations against all control treatments or standard treatment, emphasizing treatment evidence and promising responses in the literature. We believe it is useful to assess the quality of these studies and to evaluate the efficacy and safety of the data provided by the tests in terms of principles and measures of evidence-based medicine.

## 2. Methods

### 2.1. Search Strategy for the Identification of Studies

A computerized search in three electronic databases and the hand search performed by reviewing the references of the included articles resulted in nine studies meeting the inclusion criteria. Two raters independently extracted data and rated the trials for quality. The following electronic databases were assessed (performed between November/2012–January/2013) for publications dated between 1964 and December/2012: Medline, Embase, and Scopus. Free text searches were performed across each database to combine the terms or key words: “fibromyalgia” and “medicinal plants” and “treatment.” The general structure of the search strategy was “fibromyalgia” with the following MeSH terms or synonyms: (Fibromyalgias) OR (Fibromyalgia-Fibromyositis Syndrome) OR (Fibromyalgia Fibromyositis Syndrome) OR (Fibromyalgia, Primary) OR (Fibromyalgias, Primary) OR (Primary Fibromyalgia) OR (Primary Fibromyalgias); “Plants, medicinal” MeSH terms or synonyms were: (Medicinal Plant) OR (Plant, Medicinal) OR (Medicinal Plants) OR (Medicinal Herbs); and “Treatment” MeSH terms or synonyms were (Outcome, Treatment) OR (Treatment Effectiveness) OR (Effectiveness, Treatment) OR (Treatment Efficacy) OR (Efficacy, Treatment).

### 2.2. Inclusion and Exclusion Criteria

RCTs comparing herbal preparations versus placebo or other drugs administered either orally or topically in the treatment of patients with FM were included. At least one of the outcomes should be pain, sleep, quality of life, physical function, or anxiety. Only papers in English were included. We excluded case reports, researches on acupuncture, moxibustion, cupping, and articles on complementary or alternative medicine (CAM) therapies that did not include plants in their comments and literature reviews. 

### 2.3. Data Extraction, Quality Assessment, and Risk of Bias

All articles were read, and data were extracted from the articles based on predefined selection criteria by two independent reviewers. The methodological quality of the included RCTs was evaluated by using both JADAD scale [23] and the “Risk of Bias table” recommended by Cochrane Collaboration [24].

## 3. Results

### 3.1. Studies Description

**Figure 1:**
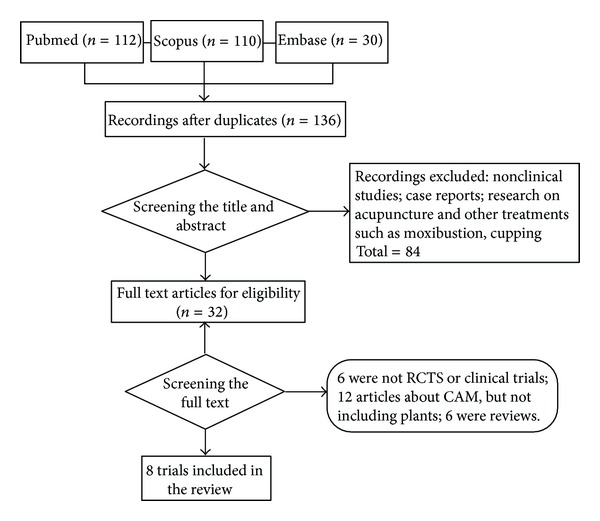
Flow diagram of the literature search.

The literature search yielded 252 results and 220 were excluded, leaving us with 32 eligible full-text articles. Out of these, 24 more were excluded and eight RCTs remained (Figure 1). Characteristics of included studies and quality assessment according to JADAD quality scale [23] are summarized in Table 1. A placebo procedure was employed in all eight trials [15–22].

The ratio of male/female participants in five studies was 19/202. Three studies [16, 17, 21] did not report gender distribution. A total of 475 subjects were included in the eight studies. The ages of the patients were 18–79 years and the average size of the trials was 29–133 participants.

The inclusion criteria for FM in all studies reviewed were the diagnostic criteria for FM, established in 1990 by the American College of Rheumatology and actualized for Wolfe et al. (2010) [25], which includes widespread pain for at least three months and tender point with 4 kg of pressure at 11 or more of 18 characteristic sensitive points [26–28]. 

Five interventions were administered orally [17, 18, 20–22] and three topically [15, 16, 19]. The duration of study varied from one day to twelve weeks, one to four times per day. The sample size also varied from 29 to 133, totaling 475 patients.

The main variables measured are related to the signs and symptoms of fibromyalgia. As measured results, the pain was assessed in all studies, with significant pain intensity reduction in five of those studies [15, 16, 18, 21, 22]. Other variables were assessed: quality of life [17, 18, 22], physical performance [20], anxiety [21], and sleep disturbance [22]. All variables were measured using internationally validated instruments (Table 1).

Adverse effects related to the use of plants in FM patients included transient burning and pricking at the application site [18], dizziness, nausea, dry mouth, drowsiness, constipation, insomnia [15], skin irritation [20], moderate transient stinging, or burning at the application sites, which generally decrease or disappear throughout the treatment.

The most common reasons for withdrawal, dropout, and/or loss during followup were personal reasons or adverse events [15–18, 22] and stigma associated with the use of cannabinoids or previous oral use [21]. Other studies did not report the reasons for discontinuation of treatment [19, 20].

### 3.2. Risk of Bias in Included Studies

**Figure 2:**
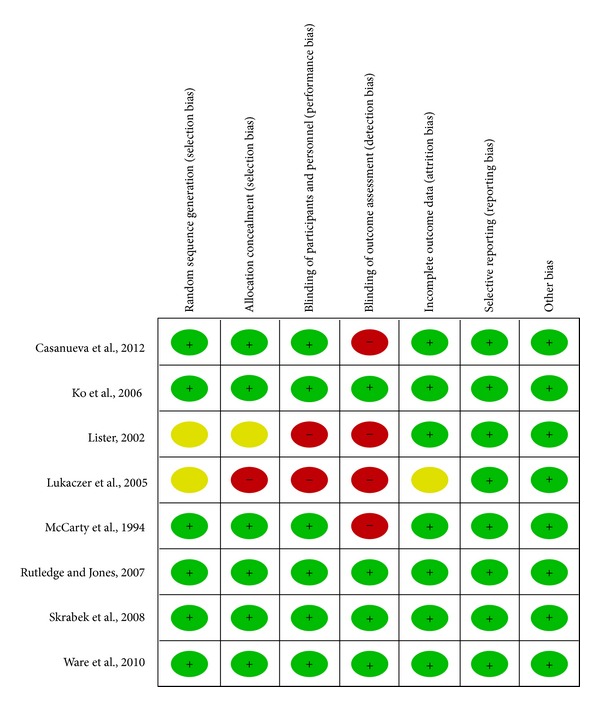
Risk of bias summary: review authors' judgments about each risk of bias item for each included study. Green: unclear risk of bias; yellow: uncertain risk of bias; red: high risk of bias.

**Figure 3:**
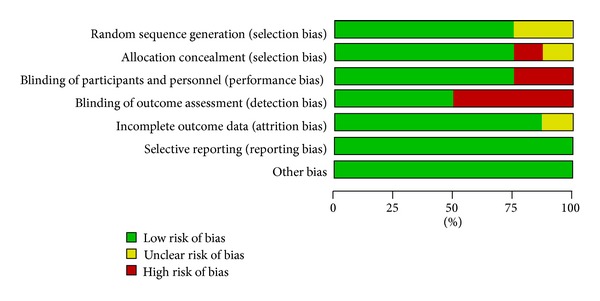
Risk of bias graph: review authors' judgments about each risk of bias item presented as percentages across all included studies.

The methodological quality of each study is summarized following Cochrane Handbook recommendation [24]. Author's judgment about “methodological quality” for each included study and percentages across all included studies are shown in Figures 2 and 3, respectively.

Three studies [20–22] reported using a random number table, one study [15] reported using a computer-generated random-number table, another [16] reported using newspaper advertisements and internet communication to FM support groups, and three studies [17–19] used no allocation concealment and thus presented a high risk of selection bias.

Five studies [16, 19–22] were double-blinded; thus, these studies had a low risk of performance bias and low detection bias. The other three studies [15, 17, 18] did not use blinding methods and had a high risk of performance bias or a strong detection bias.

No detailed evidence of selective reporting was found in any of the eight studies. As for other potential sources of bias, one study exhibited a patient compliance, which could suggest acceptance and applicability of the product.

### 3.3. Effects of Interventions

All studies compared MP or RNP basic therapy to standard treatment or placebo. The medicinal plants treatments included the Modified Meta050 [18], provided as a tablet containing 440 mg of a proprietary formulation of RIAA (as magnesium salt from *Humulus lupulus* cone extract), standardized rosemary extract (*Rosmarinus officinalis*), and oleanolic acid (from *Olea europaea* olive leaf extract). Another treatment was coenzyme Q10 [17], provided in gelatine capsules and 100 mg tablets of *Ginkgo biloba* standardized to contain 24 mg flavone glycosides and 6 mg terpene lactones (Bio-Biloba, pharma Nord). Among RNPs, two studies used nabilone [21, 22]; among MPs two used capsaicin [15, 19], and two used O24 [16, 20].

Two studies investigated the effects of topical capsaicin, a compound obtained from chili peppers (*Capsicum annuum* L.), in FM patients. An RCT tested the efficacy of the local application of 0.075% capsaicin in FM patients [15] compared with patients from the control group, who continued taking the usual treatment provided before randomization. At the end of the intervention, 108 patients were assessed for pain, which was related to other clinical outcomes: body mass index (BMI) and the number of physical symptoms, myalgic score, pressure pain threshold, pain, fatigue, anxiety, and depression. Capsaicin-treated patients showed improvement in myalgic score and in the end of the therapy patients of both groups presented a global subjective improvement that was defined as decrease in pain, improvement in physical function, and in sleep or fatigue. At the end of the followup, six weeks after capsaicin discontinuation, those who were treated with topical capsaicin still showed significant improvement in several clinical outcomes compared to controls—namely, myalgic score, pressure pain threshold, FSS, FIQ, VAS of depression, and role limitations due to emotional problems.

Another double-blinded RCT [19] that investigated the effects of topical capsaicin tested the efficacy of the local application of 0.025% capsaicin in the treatment of FM, comparing with placebo cream and control group. Forty-five patients were randomly assigned, and after four weeks of double-blinded treatment, they were assessed for pain, stiffness, and sleep disruption. Significant improvement of sensitivity and pain was associated to capsaicin. However, there was no improvement in pain or quality of sleep.

Two studies investigated the effects of oral nabilone, a synthetic cannabinoid in FM patients. One study [22] was a randomized, double-blinded, active-control, equivalency crossover trial to compare nabilone to amitriptyline, before bedtime in patients with FM reporting chronic insomnia. Twenty-nine subjects received each drug for two weeks with a two-week washout period and were assessed for sleep quality, pain, quality of life, and adverse events. Nabilone was effective in improving sleep in patients with FM and was well tolerated, which may be considered an alternative strategy to amitriptyline. The effects of nabilone on pain, mood, and quality of life were similar to those seen with amitriptyline. Adverse effects were more common with nabilone, particularly drowsiness and dizziness, as are common for other cannabinoids, although global satisfaction with both drugs was similar.

One randomized, double-blinded, placebo-controlled trial [21] aimed at determining the benefit of nabilone in pain management and life quality improvement in FM patients. Forty patients were randomized and received nabilone, from 0.5 mg at bedtime to 1 mg over 4 weeks or a corresponding placebo, and after four weeks were reassessed for pain and the number of tender points. There were significant decreases in pain and anxiety in the nabilone-treated group in four weeks. There were no significant improvements in the placebo group. Nabilone appears to be a beneficial, well-tolerated treatment option for FM patients, with significant benefits in pain relief and functional improvement.

The O24 Pain Neutralizer, a natural pain relief solution which contains camphor oil (white) (Japan), eucalyptus oil (Australia), *Aloe vera* oil (Mexico), peppermint oil (India), rosemary oil (Spain), lemon oil (USA), and orange oil (USA), was investigated by two studies for its possible effects in FM patients. The study of Rutledge and Jones (2007) [20], a randomized clinical trial, compared O24 with sham oil combined with exercise, and 43 women were trained in O24 application before exercise, at bedtime on exercise days. After a twelve-week period, the patients were assessed for exercise volume, pain, physical performance, and physical function. There was no significant difference in exercise volume between women using O24 as compared with that using sham oil after twelve weeks. The authors concluded that increases in physical function found, while not significant, may be attributable to the exercise regimen or to the interaction of the oils and exercise regimen. 

One double-blinded placebo-controlled trial study compared O24 with placebo [16], and 153 subjects were randomized and trained in O24 application every four hours as needed for pain, or placebo oils (peppermint oil) identical in smell and appearance, and active oils were supplied to the other half. After four weeks, patients were assessed for pain, tender point, and strength. With topical O24 over the placebo, improvements were noted in the visual analog scale night pain rating.

Meta050, a proprietary standardized combination of reduced iso-alpha acids from hops, rosemary extract, and oleanolic acid, were investigated for their possible effects on pain in patients with rheumatic disease: osteoarthritis, rheumatoid arthritis, and FM [18]. Fifty-four patients received 440 mg of Meta050 three times a day for four weeks, which was changed to 880 mg twice a day for the subsequent four weeks in the majority of patients. We analyzed pain and specific symptoms by means of the Abridged Arthritis Impact Measurement Scale (AIMS2) and the Fibromyalgia Impact Questionnaire (FIQ). FM patients demonstrated some improvement on pain; however, the decrease in pain did not reach significance within four weeks. No significant difference was observed in grip strength on these subjects, either. In general, pain and stiffness were moderately improved, but only after eight weeks on the supplement.

One open clinical trial was undertaken to measure the subjective effects of coenzyme Q10 combined with a *Ginkgo biloba* extract in volunteer subjects with clinically diagnosed FM [17]. Thus, 23 subjects received oral doses of 200 mg coenzyme Q10 combined with 200 mg *Ginkgo biloba* extract. After twelve weeks, patients were assessed for quality of life; 68% of those who completed the trial expressed the view that they would like to continue with treatments. The overall subjective views of the patients, regardless of quality of life scores, were that the majority (64%) felt the treatment was of some benefit.

### 3.4. Quality of Evidence

The methodological quality of all included trials was evaluated by using JADAD scale and two studies were rated as good quality [15, 21]; four studies, moderate [16, 19, 20, 22]; and two studies, low [17, 18].

## 4. Discussion

Considerable efforts have been made to discover new analgesic agents with increased efficacy and fewer side effects. Therefore, our review aimed at investigating the effects of MP or RNP in FM patients, based on an analysis of eight RCTs, trying to propose new possibilities that can offer secure relief in symptoms. Interestingly, the results found indicated similar pharmacological effects between related products or MP natural and standard drugs used in controlling symptoms of FM. When compared with placebo, MP or RNP demonstrated positive effects in pain relief, quality of life, anxiety, sleep disruption, and physical function. The clinical challenge persists because there is not yet a specific target with therapeutic applicability.

This challenge also affects the drugs that are already part of the clinical management of FM. Although drug therapy is very broad and added the clinical approach of these patients, currently, duloxetine, pregabalin, and milnacipran are the only ones approved by the FDA for the treatment of FM [29]. The mechanisms of action of pregabalin, duloxetine, and milnacipran are related to the pathophysiology of FM; however, these therapeutic agents are not effective for all patients with FM. A recent study by Katz et al. suggests that the diagnostic criteria for fibromyalgia may be partly responsible for the fact that there is currently no gold standard for the diagnosis and treatment of FM [30].

In this review, we found two studies involving capsaicin in the treatment of FM [15, 19]. Capsaicin, the main active capsaicinoid ingredient of chili peppers (*Capsicum* spp.), an agonist of the transient receptor potential vanilloid 1 (TRPV1) receptor [31], is highly expressed on nociceptors [32–34], which causes an initial excitation of the neurons and a period of enhanced sensitivity. This selective stimulation of afferent C fibers and the release of substance P (SP) are followed by a refractory period with reduced sensitivity. Repeated applications lead to persistent desensitization possibly due to depletion of SP at nervous afferent endings and transiently decrease the density of nervous fibers on the skin [35]. Repeated applications lead to a long-lasting desensitization towards pain due to the increase in pain threshold and a flare on the skin in FM patients, suggesting an increased activity of polymodal nociceptors.

Recent evidence demonstrates that the vanilloid receptor, stimulated by capsaicin or by endocannabinoids in ventral periaqueductal gray (vPAG), induces analgesia [36]. This analgesic effect is associated with increased release of glutamate and activation of rostral ventromedial medulla cells. The activation of the descending nociceptive stimulation of this receptor in the vPAG may be a new strategy for producing analgesia [37, 38]. Given the benefits of using capsaicin, which was also approved by the FDA for the management of neuropathic pain in November 2009 [32], the adverse effects are manageable and do not constitute a limitation to its use, but a motivation of new formulations that may have better applicability and acceptance. 

Although pain is the primary chronic symptom, disturbed sleep is also a major symptom of patients with FM; patients report difficulty falling asleep, significantly more nighttime awakenings, and unrefreshing sleep [39, 40]. Sleep problems have been related to both depression and pain among patients with FMS in some studies [38, 40] and insomnia has been frequently reported in FM patients [41]. 

Endogenous cannabinoids have been postulated to have an effect on normal sleep induction [42]. Nabilone, a synthetic cannabinoid, has been observed in a small case series to improve sleep in patients with chronic pain [41]. Researchers conducted clinical trial of cannabinoids for other chronic pain disorders and have reported benefits on sleep as secondary outcome [41]. One of the studies included in this review [22] confirmed the indirect positive effect on sleep as it was observed that both synthetic cannabinoid nabilone and amitriptyline, a tricyclic antidepressant, had a favorable effect on sleep in patients with FM, with nabilone showing higher overall superiority than amitriptyline in relation to the quality of sleep.

Although there are no specific clinical studies on the use of cannabinoid receptor agonists for symptomatic relief of FM, some findings support their therapeutic potential due to their anti-inflammatory, analgesic, and sedative properties [42]. The analgesic benefits of cannabinoids in the treatment of acute and chronic pain have already been suggested [43], and according to another study that was assessed, pain and anxiety symptoms were attenuated using cannabinoid [21]. This benefit was probably secondary to a clinical endocannabinoid deficiency in FM patients as has been suggested [41] or the synergistic relationship with endogenous opioids [44]. Behavioral studies provide evidence that both CB1 and CB2 receptors can contribute to muscular antinociception and, interestingly, suggest that the local administration of CB agonists could be a new and useful pharmacological strategy in the treatment of muscular pain, avoiding adverse effects induced by systemic administration [45].

A pilot RCT study performed by Traitses et al. (2007) [46] suggests that FM patients may be effectively and safely managed for pain with topical O24. Two studies reported benefits after treatment with O24, a topical pain relief agent [16, 20]. Compared to other topical products, O24 is unique in incorporating well-studied botanicals without alcohol, glycerin, synthetics, or preservatives. Each active ingredient of O24 (camphor oil, eucalyptus oil, aloe vera oil, peppermint oil, rosemary oil, and lemon and orange oils), has been studied for pain [47–49] and the primary mode of action is a counterirritant for pain sensation and the local effects include inhibition of pain transmitters such as bradykinin, histamine, and prostaglandins [50].

Another RCT used the same agent to verify the viability of topical O24 in FM patients, associated with exercise. In previous studies, da Costa et al. found that pain and fatigue decrease in activity for subjects with FM and chronic fatigue syndrome, rather than bouts of exercise leading to pain and fatigue [51]. Muscle stretching generates a positive impact in the treatment of fibromyalgia, improving pain, stiffness, and quality of sleep [52], and the aerobic activities were considered effective, including benefits months after the end of treatment [53]. Thus, a double-blind RCT that assessed the joint action of O24 associated with an exercise program [20] observed benefits in physical function and pain relief in FM patients which enabled greater adherence to exercise program.

The prevalence of psychological abnormalities, particularly depression, is high among FM patients [54–56]. Meta 050, a tablet containing 440 mg of a proprietary formulation of RIAA (as magnesium salt from Humulus lupulus cone extract), standardized rosemary extract (*Rosmarinus officinalis*) and oleanolic acid (from Olea europaea olive leaf extract). Rosemary, *Rosmarinus officinalis* L., has many therapeutic applications in popular medicine to cure or manage a wide range of diseases, including depression [57]. The oleanolic acid (OA) is a pentacyclic triterpene that pertains to oleanane series naturally found in various medicinal herbs traditionally used for anti-inflammatory, analgesic, hepatoprotective, and cardiotonic effects [58]. The antinociceptive effect of OA appears to involve endogenous opioids as it was blocked in mice pretreated with naloxone, a nonselective opioid antagonist; this supports the notion that compounds like OA might be useful as pain relievers [59]. The study assessed in this review [18] supports previous conclusions and suggests that Meta050 can offer moderate benefits for the management of pain and depression.

Given the vast symptomatology that is present in FM, the quality of life could not change coursing with large negative impact on the lives of these patients [60, 61]. Moreover, several studies show that there are significant changes in the balance oxidant/antioxidant in triggering the FM, with a significant increase in the number of free radicals [62–64]. Some FMS patients with muscle pains have found benefit from coenzyme Q10, while others with central nervous system symptoms have found benefit from *Ginkgo biloba* extract. Coenzyme Q10 is essential for normal muscle activity and a deficiency in this coenzyme is thought to impair function [65]. *Ginkgo biloba* extract can improve vascular function in both muscle and brain [66]. As both of these agents are antioxidants and free radical scavengers, it is possible that the benefit demonstrated in a study assessed in this review [17] may be due, at least in part, to an antioxidant activity.

The methodological limitations of the studies reported in this systematic literature review included small sample sizes, lack of blinding, and differences in treatment as well as in the severity of disease of the participants, are also factors that potentially introduce difficulties in the analysis, as large differences in treatment effects can be expected. Although strong efforts were made to retrieve all RCTs on the subject, we cannot be absolutely certain that we succeeded.

The results also may have been compromised by heterogeneity. The chemical composition of the weed grown naturally varies according to weather conditions, harvest time, storage conditions, and so forth. This variability can result in significant differences in pharmacological activity, making it difficult to standardize them botanically [67]. Studies using standard formulations also show interesting activity that was isolated from each of the components of the formula. Due to the limited number of RCTs included, we cannot draw definitive conclusions about MP or RNP in the treatment of patients with FM; however, the results allow us to believe in the potential of plants as biological targets to be studied in order to achieve better therapeutic action and fewer adverse effects.

## 5. Conclusion

Based on the current review, it is unclear whether MP or RNP is effective in treating fibromyalgia. However, it was noted that these therapies are promising in the treatment of rheumatic conditions as chronic fibromyalgia. More studies with adequate methodological quality in order to investigate the efficacy and safety of MP or RNP for fibromyalgia are needed.

## Figures and Tables

**Table 1:** Summary of randomized clinical studies of medicinal plants or related natural product in fibromyalgia.

Reference/year	Plant/route	Sample size	Duration of study	Outcome measured	Clinical assessment instrument	Findings of the study	Adverse effects	Jadad score
Casanueva et al., 2012 [15]	Capsaicin/topical	108	12 weeks	Pain Fatigue Anxiety Depression	VAS BPI MPQ FIQ FSS	Significant improvement in pain, depression, fatigue	Transient burning and pricking	5

Ko et al., 2007 [16]	Oil 24/topical	133	one month	Pain Subjective surveys of pain Measurements of tender point	VAS FIQ FDK 10 TePs	Significant improvement in surveys of pain and in measures of tender point	Skin irritation	4

Lister, 2002 [17]	Coenzyme Q10 and Ginkgo biloba/oral	23	12 weeks	Quality of life	SF-36	Significant improvement in quality of life	Little or no clinical significance	1

Lukaczer et al., 2005 [18]	Meta050/oral	54	8 weeks	Pain Quality of life Physical function	VAS FIQ SF-36 PCS MCS	Significant improvement in pain	No evidence suggested	1

McCarty et al., 1994 [19]	Due capsaicin/topical	45	4 weeks	Pain Tenderness Stiffness	FIQ VAS TePs	Significant improvement in pain, tenderness, and stiffness	Moderate transient stinging or burning	3

Rutledge and Jones, 2007 [20]	Oil 24/topical	43	12 weeks	Pain Physical performance Exercise volume Physical function	BPI GSI BMI HGS FDK	Significant improvement in physical function	No evidence suggested	4

Skrabek et al., 2008 [21]	Nabilone/oral	40	4 weeks	Pain Anxiety	VAS FIQ	Significant improvement in pain and anxiety	No evidence suggested	5

Ware et al., 2010 [22]	Nabilone/oral	29	10 weeks	Sleep disruption Pain Quality of life Mood	ISI LSEQ MPQ SF-36 FIQ HADs-D	Significant improvement in quality of sleep	Dizziness subjects, nausea, dry mouth, drowsiness, constipation Insomnia	4

BMI: Body Mass Index. BPI: Brief Pain Inventory. FIQ: Fibromyalgia Impact Questionnaire. Force Dial FDK 40: Standard Pressure Algometry. FSS: Fatigue Severity Scale. GSI: Global Severe Index. HADs-D: Hospital Anxiety and Depression Scale. HGS: Handgrip Strength. ISI: Insomnia Severity Index (ISI); LSEQ: The Leeds Sleep Evaluation Questionnaire. MCS: Mental Function Score. MPQ: The McGill Pain Questionnaire. PCS: Physical Function Score. SF-36: Quality of Life-Short-Form 36; TePs: Pressure Algometry Measurements of Tender Point. VAS: Visual Analog Scale. VASs: Visual Analogue Scale.

## Abbreviations

BPI: Brief Pain Inventory
BMI: Body Mass Index
CAM: Complementary and alternative medicine
FDA: Food and Drug Administration
FDK: Force Dial—Standard Pressure Algometry
FIQ: Fibromyalgia Impact Questionnaire
FM: Fibromyalgia
FSS: Fatigue Severity Scale
GSI: Global Severe Index
HAD: Hospital Anxiety and Depression Scale
HGS: Handgrip Strength
ISI: Insomnia Severity Index
LSEQ: The Leeds Sleep Evaluation Questionnaire
MCS: Mental Function Score
MPs: Medicinal plants
MPI: Multidisciplinary Pain Inventory
MPQ: McGill Pain Questionnaire
NSAIDs: Anti-inflammatory nonsteroidal drugs
OA: Oleanolic acid
PCS: Physical Function Score
PHN: Postherpetic Neuralgia
PSQI: Pittsburgh Sleep Quality Index
QLS: Quality of Life Questionnaire
RCTs: Randomized controlled trials
RNPs: Related natural products
SCPV: Ventral periaqueductal Gray
SF 36: Short Form 36 Health Survey
SRs: Systematic reviews
TePs: Pressure Algometry Measurements of Tender Point
VAS: Visual Analog Scale
vPAG: Ventral periaqueductal gray.
